# Multi-genomics data mining identified critical transcription factors and immune response pathways in traumatic optic nerve injury and retinal injury

**DOI:** 10.3389/fopht.2026.1762890

**Published:** 2026-07-27

**Authors:** Jinjun Li, Yong Zhang, Shu Zhou

**Affiliations:** Department of Emergency, The People’s Hospital of Liuyang, Changsha, China

**Keywords:** hub genes, immune response, regulatory mechanism, retinal ganglion cells, retinal injury, traumatic optic nerve injury

## Abstract

**Background:**

Traumatic optic nerve and retinal injuries can lead to severe visual impairment, with undetermined underlying molecular mechanism and few reliable molecular biomarkers. This study aims to identify dysregulated genes and signaling pathways potentially involved in these injuries.

**Methods:**

We first identified differentially expressed genes (DEGs) and their enriched functions using two time-course gene expression profile datasets after retinal injury of rats. The hub genes were then obtained from DEGs by protein-protein interaction network, and then assessed by receiver operating characteristic curve analysis to check their diagnostic potential in experimental datasets. The potential miRNAs and transcriptional factors (TFs) of hub genes were also investigated.

**Results:**

We identified 312 and 262 DEGs from the two datasets, respectively, with 31 overlapped up DEGs in injury group. These co-up DEGs were highly enriched in immune response-associated pathways and visual system development pathways, including the pivotal JAK-STAT signaling pathway. We then identified 7 hub genes, of which six (*Aif1*, *Cntf*, *Gfap*, *Lcn2*, *Stat1*, and *Stat3*) were validated to be associated with diagnostic relevance in experimental datasets. We predicted 54 miRNAs and 25 TFs participating in the regulatory network of hub genes. Finally, we identified that STAT1 and STAT3 were potential transcriptional regulators in hub genes.

**Conclusion:**

We identified six critical hub genes and potential regulators that may participate in traumatic optic nerve injury, extending the underlying mechanisms of retinal injury.

## Introduction

Ocular trauma, including traumatic optic nerve injury (TONI) and traumatic retinal injury (TRI), is a critical condition frequently encountered in emergency departments. The failure to perform prompt and effective treatment of ocular trauma can lead to permanent vision impairment or blindness. According to previous literature, the incidence rate of traumatic optic neuropathy is approximately 0.5−5.0% (1, 2). Retinal ganglion cells (RGCs) are specific projective neurons involved in transmitting visual signals from the retina to the brain (3). The optic nerve comprises RGC axons. Various types of optic neuropathy, such as trauma (4), glaucoma (5), diabetic retinopathy (6), optic neuritis (7), and ischemic optic neuropathy (8), can result in the degeneration or death of RGCs, leading to visual impairment. However, the specific molecular and cellular mechanisms responsible for RGC death remain unelucidated, and effective diagnostic and therapeutic targets are lacking.

Following rapid advancements in high-throughput omics techniques, particularly microarray and transcriptome sequencing (RNA-seq), these methods have been increasingly applied to ophthalmic research, enabling the genome-wide profiling of gene expression changes under various pathological conditions (9, 10). By utilizing bioinformatics methods, the whole genomic gene expression profiles can be analyzed and processed, thereby providing valuable gene targets for disease diagnosis, treatment, and prognosis (11). Currently, many studies have utilized global gene expression datasets to identify critical genes and investigate their molecular mechanisms associated with ophthalmic diseases, such as myopia (12), glaucoma (13), and diabetic retinopathy (14). While a few studies have reported temporal gene expression changes following optic nerve or retinal damage, most have focused on descriptive differential expression without integrating cross-dataset validation, protein-protein interaction networks, or upstream regulatory analyses.

In this study, we aimed to systematically investigate gene expression profiles and identify potential hub genes and signaling pathways that participate in inducing TONI or TRI. By analyzing two time-course gene expression profiles after optic nerve and retinal injury, including GSE9918 (15) and GSE1001 (16), we screened out the DEGs and the co-expressed DEGs to obtain candidate genes, which were then analyzed to identify their functions and interaction network, as well as the upstream regulators of hub genes. In summary, our results extend the underlying regulatory mechanisms of TONI and TRI, and identify the potential hub genes that can be further validated to check their potential as diagnostic or therapeutic candidates in future.

## Materials and methods

### Microarray data acquisition

We extracted two microarray gene expression datasets from the GEO database, including GSE9918 and GSE1001. GSE9918 contained 5 control samples and 15 samples from rats with intra orbital nerve crush, while GSE1001 included 3 normal samples and 15 samples from rats with retinal tears induced by scraping the retina with a needle. The detailed information of these two datasets was shown in **Table 1**.

**Table 1:** Details of GSE9918 and GSE1001 datasets, including sample source, sample number, and platform.

Animal	Model	Sample	Dataset	Platform	Injury	Control
Sprague-Dawley rats	IONC	Retina	GSE9918	GPL1355	15	5
Sprague-Dawley rats	Retinal tear	Retina	GSE1001	GPL85	15	3

IONC, intra-orbital nerve crush.

### Identification of DEGs

We identified DEGs between the control and injury groups in each dataset by using the “limma” (version 3.52.2) package (17) of R (version 4.2.1) software. The thresholds for screening significant DEGs were as follows: |log_2_(fold change)| > 1.0; adjusted *p*-value (or *p*-value) < 0.05 by false discovery rate (FDR) correction. DEG expression was visualized with the “ggplot2” (version 3.3.6) package (18) and “ComplexHeatmap” (version 2.13.1) package (19). Venn diagrams were created to identify co-expressed DEGs from GSE9918 and GSE1001 with the “ggplot2” package and “VennDiagram” (version 1.7.3) package.

### GSEA

GSEA enables to reveal gene expression patterns related to specific biological processes, pathways, or functions. It assesses the distribution trends of predefined gene sets in the gene expression data to determine how each gene set contributes to the phenotype. We utilized “clusterProfiler” (version 4.4.4) package (20) to perform GSEA. The cutoff criteria were as follows: false discovery rate (*q*-value) < 0.25; adjusted *p*-value < 0.05. The “ggplot2” package was utilized for visualizing the results.

### GO and KEGG analyses

Biological functions of the co-expressed DEGs were determined by GO analysis for three categories: biological processes, cellular components, and molecular functions. KEGG analysis was utilized for assessing the role of the co-expressed DEGs in the signaling pathways. Both KEGG and GO analyses were carried out using the “clusterProfiler” package, with statistical threshold at *p* < 0.05. Enrichment results were depicted with the “ggplot2” package.

### PPI network construction and hub gene selection

To assess interactions among the co-expressed DEGs, we developed a PPI network with the online STRING database (https://cn.string-db.org/) (version 12.0). “*Rattus norvegicus*” and “medium confidence (0.400)” were set as species and the minimum required interaction score, respectively. Subsequently, by using Cytoscape (version 3.8.2) software, we constructed and visualized the PPI network. The potential hub genes were screened with cytoHubba and MCODE plugins.

### Validation of the hub genes

The expression matrix of the potential hub genes was obtained from GSE9918 and GSE1001 and compared between the different groups by using the “car” (version 3.1-0) and “stats” (version 4.2.1) packages. Genes showing remarkable differences in their expression patterns in both datasets were selected for further analysis. Subsequently, ROC curves were generated with the “ggplot2” and “pROC” (version 1.18.0) packages to confirm the diagnostic relevance of these hub genes within experimental datasets.

### miRNA and transcription factor analysis

To investigate functions of the hub genes in human ocular trauma diseases, we performed homologous transformation of rat genes to human genes by using the “homologene” package (version 1.4.68.19.3.27). We utilized the miRTarBase (https://mirtarbase.cuhk.edu.cn/) database to identify miRNAs linked with the hub genes. Furthermore, we explored the hTFtarget (https://guolab.wchscu.cn/hTFtarget/) database and JASPAR (https://jaspar.elixir.no/) database to determine transcription factors (TFs) related to the hub genes. Cytoscape software was utilized to visualize the results.

## Results

### DEG analysis demonstrated were 31 co-upregulated DEGs during the process of TONI and TRI

**Figure 1:**
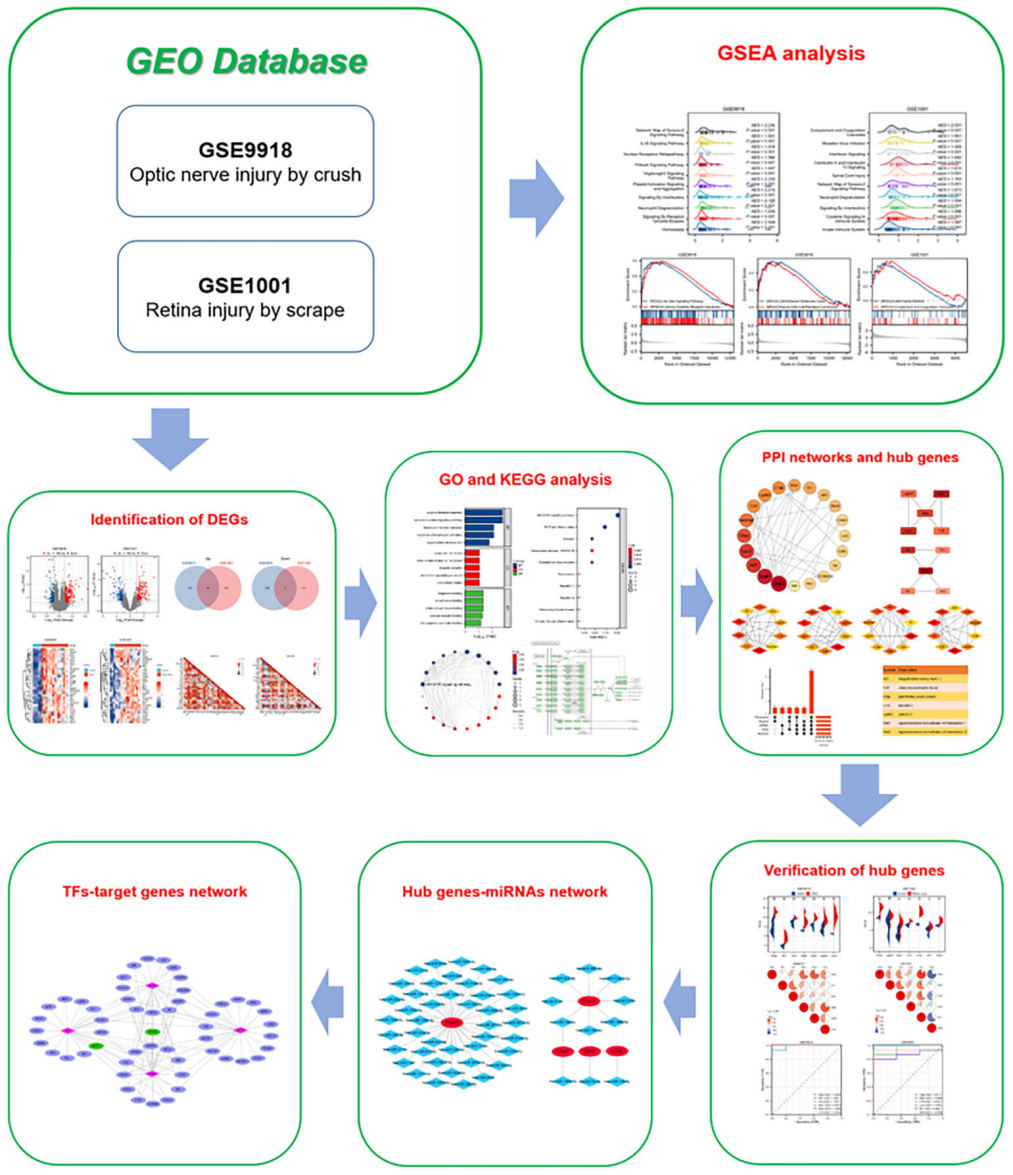
The flow chart for the comprehensive analysis of this study. Two datasets were employed to obtained the DEGs and following enriched pathways. Regulatory network was finally predicted based on the identified hub genes. GSEA, Gene Set Enrichment Analysis; DEGs, Differentially Expressed Genes; GO, gene ontology; KEGG, Kyoto Encyclopedia of Genes and Genomes; PPI, protein-protein interaction; ROC, receiver operating characteristic; TF, transcription factor.

**Figure 2:**
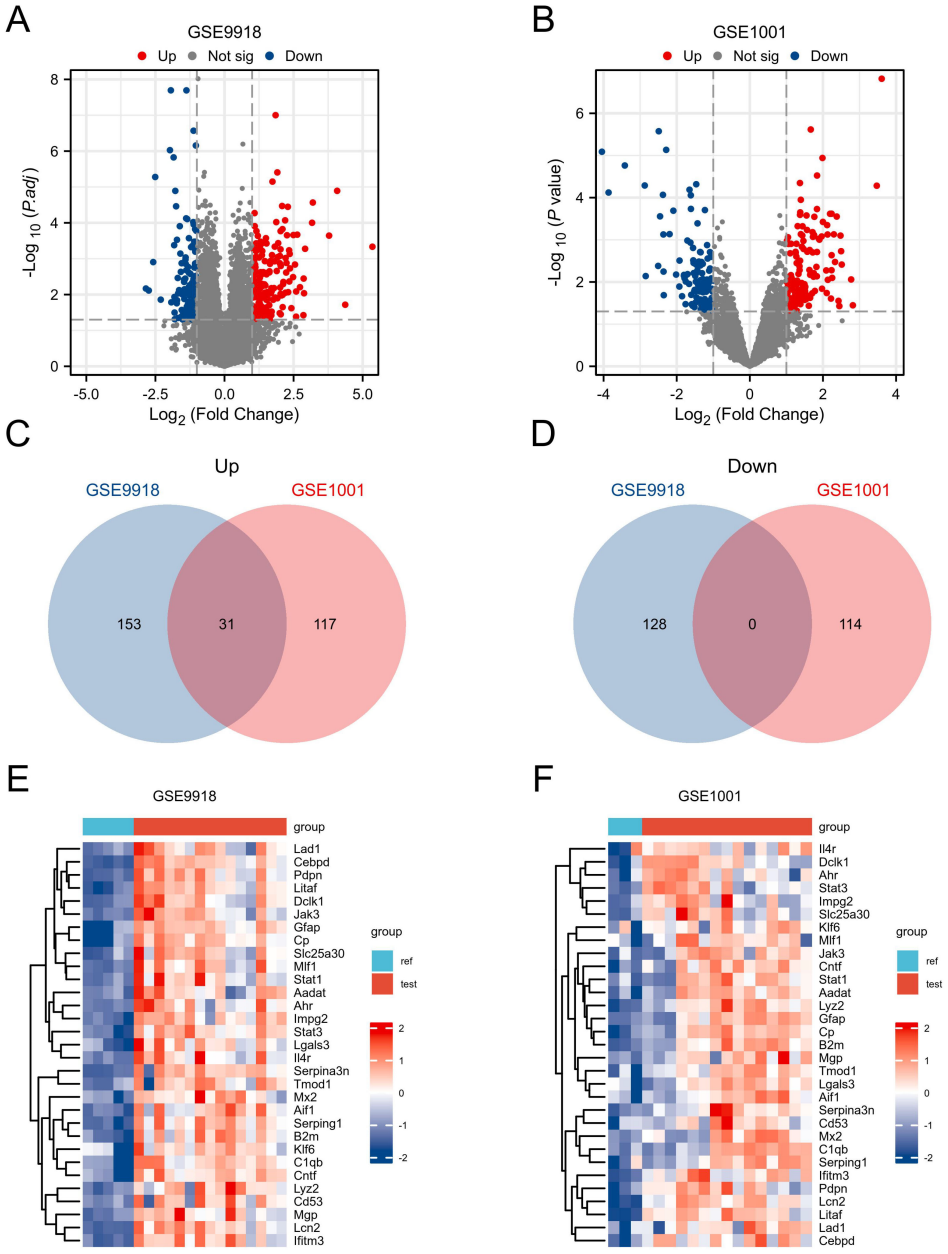
DEG analysis identified hundreds of DEGs from these two datasets and overlapped DEGs. **(A, B)** Volcano plots depicting the expression and identification of DEGs in the GSE9918 and GSE1001 datasets, respectively. **(C, D)** Venn diagrams showing the overlapped DEGs within the upregulated and downregulated DEGs in GSE9918 and GSE1001 datasets. **(E, F)** Heatmaps showing the expression pattern of the 31 co-expressed up DEGs in GSE9918 and GSE1001.

**Figure 3:**
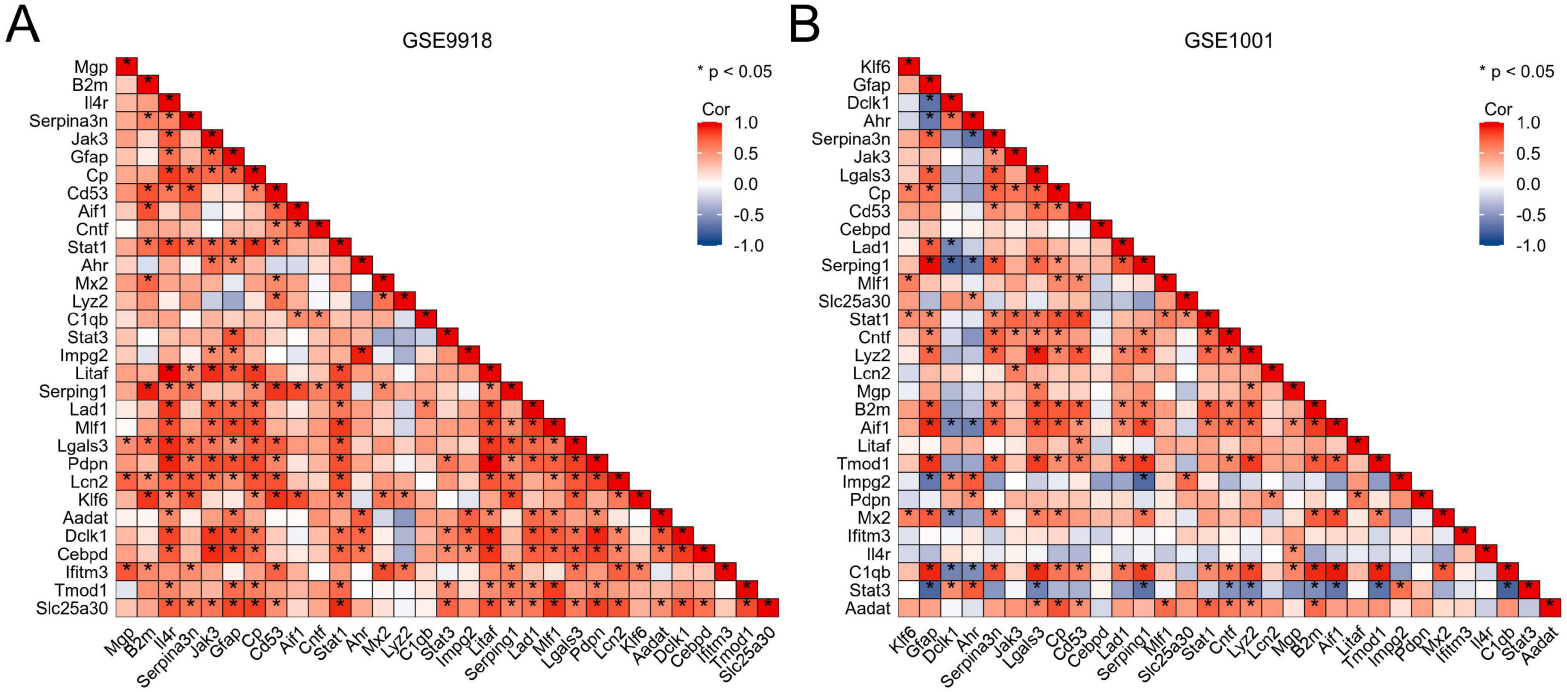
Correlation analysis of the 31 co-up DEGs in the two datasets. **(A, B)** Heatmap showing the Spearman’s correlation coefficients of the 31 co-up DEGs in GSE9918 (left panel) and GSE1001 (right panel). Red and blue points denote positive and negative correlations, respectively. *p < 0.05.

To identify the potential regulators of TONI or TRI, we explored the global gene expression profiles of samples from injured rat model, and systematically investigated the regulated DEGs after injury, and their functions, diagnostic value, and potential upstream regulators (**Figure 1**). We identified 312 DEGs in GSE9918, including 184 upregulated and 128 downregulated genes (**Figure 2A**), and 262 DEGs in GSE1001, including 148 upregulated and 114 downregulated genes (**Figure 2B**). Venn diagrams revealed that among the overlapped DEGs between these two datasets, 31 co-upregulated DEGs were detected but no co-downregulated DEGs were detected (**Table 2**; **Figures 2C, D**). Heatmap presentation depicted the expression levels of these 31 co-up DEGs in these two datasets, showing consistent higher expression pattern in injury samples (**Figures 2E, F**). Correlation heatmaps (**Figure 3**) showed the pairwise correlation of these co-up 31 DEGs. The details of these DEGs between the two datasets were provided in **Additional File 1**. In summary, these results demonstrated that TONI and TRI can induce global gene expression changes of the retinal tissues.

**Table 2:** The list of co-up and co-down DEGs between GSE9918 and GSE1001.

Expression level	Total	Gene name
Upregulated	31	Tmod1, Mx2, C1qb, B2m, Aadat, Lcn2, Dclk1, Cebpd, Gfap, Stat1, Cd53, Impg2, Mlf1, Mgp, Pdpn, Aif1, Cp, Litaf, Klf6, Il4r, Lad1, Slc25a30, Lgals3, Ifitm3, Stat3, Lyz2, Cntf, Jak3, Ahr, Serping1, Serpina3n
Downregulated	0	—

### Immune system pathways were enriched in DEGs from both datasets

**Figure 4:**
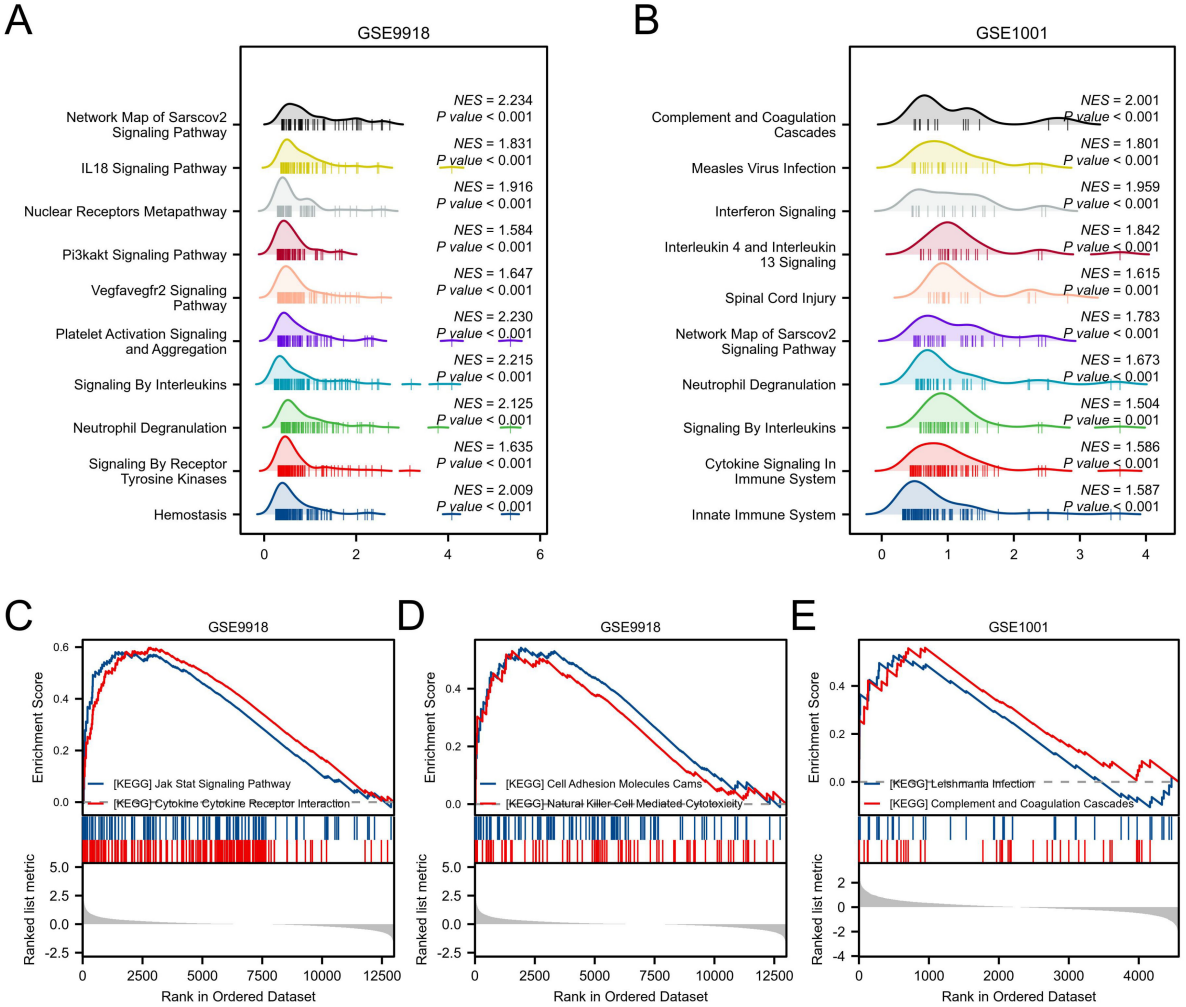
GSEA results for DEGs from GSE9918 and GSE1001. **(A, B)** GSEA-Reactome and GSEA-Wiki Pathways analyses of GSE9918 **(A)** and GSE1001 **(B)**. In the ridge plots, the x-axis and y-axis indicate log_2_FC distribution and specific biological functions or crucial signaling pathways, respectively. NES, normalize enrichment score. **(C–E)** GSEA-KEGG analysis of GSE9918 **(C, D)** and GSE1001 **(E)**. The curves represented the enriched KEGG pathways.

GSEA was then conducted to identify signaling pathways and biological functions of the DEGs from GSE9918 and GSE1001 (**Additional File 2**). By referring to the Reactome and Wiki Pathway databases, the DEGs in GSE9918 were mainly enriched in hemostasis, signaling by receptor tyrosine kinases, neutrophil degranulation, signaling by interleukins, network map of Sarscov2 signaling pathway, and IL-18 signaling pathway (**Figure 4A**). In contrast, DEGs in GSE1001 showed enrichment mainly in cytokine signaling in immune system, neutrophil degranulation, innate immune system, signaling by interleukins, and network map of Sarscov2 signaling pathway (**Figure 4B**). Based on the KEGG database, DEGs in GSE9918 were mainly enriched in JAK-STAT signaling pathway, natural killer cell-mediated cytotoxicity, cytokine receptor interaction, and cell adhesion molecules (**Figures 4C, D**), while DEGs in GSE1001 showed enrichment mainly in leishmania infection and complement and coagulation cascades (**Figure 4E**). In summary, we observed that immune system pathways were significantly dysregulated after TONI and TRI in both datasets.

### GO and KEGG analyses of co-upregulated DEGs from both datasets

**Figure 5:**
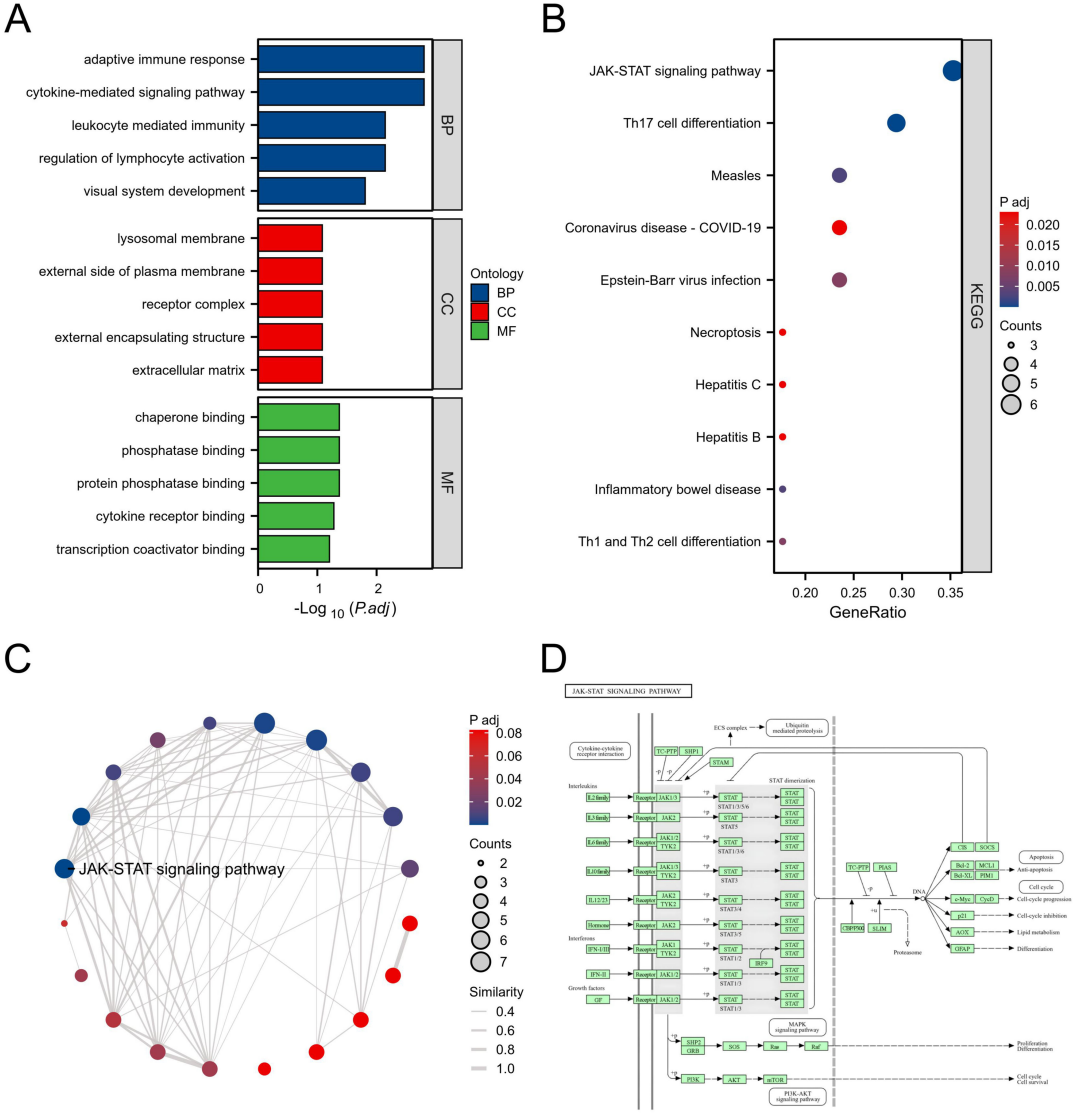
GO and KEGG enrichment analysis results of the 31 co-up DEGs in these two datasets. **(A)** GO analysis showing the top five pathways in biological processes (BP), cellular components (CC), and molecular functions (MF). **(B)** Bubble plot showing the top ten enriched KEGG pathways. **(C)** EMAP plot for the GO and KEGG analyses, indicating the importance of the JAK-STAT signaling pathway. **(D)** Signal transduction information regarding the JAK-STAT signaling from the KEGG pathway database.

Then we utilized GO and KEGG analyses to investigate functions of the 31 co-expressed DEGs (**Additional File 3**). The GO enrichment analysis demonstrated high enrichment in adaptive immune response, cytokine-mediated signaling pathway, leukocyte-mediated immunity biological processes, and extracellular matrix, external encapsulating structure, receptor complex cellular components, as well as protein phosphatase binding, phosphatase binding, cytokine receptor binding molecular functions (**Figure 5A**). KEGG analysis revealed involvement of the 31 co-up DEGs in the JAK-STAT signaling pathway and Th17 cell differentiation (**Figure 5B**). The top five enriched functions or pathways in each part were described in **Table 3**. To illustrate the relationships among the enriched GO and KEGG pathways, we constructed an enrichment map plot, indicating that the JAK-STAT signaling pathway can be a pivotal pathway (**Figure 5C**). To assess the significance of the JAK-STAT signaling pathway, we derived the pathway map and presented the signaling pathway by searching the KEGG pathway database (https://www.kegg.jp/kegg/pathway.html) (**Figure 5D**). Consistent with the GSEA results, the immune response pathways were also detected using GO and KEGG analysis methods.

**Table 3:** Table list showing the results of enriched GO and KEGG pathways.

Ontology	ID	Description	Gene ratio	Background ratio	P-value
BP	GO:0019221	cytokine-mediated signaling pathway	7/30	426/18112	0.000
BP	GO:0002250	adaptive immune response	7/30	415/18112	0.000
BP	GO:0051249	regulation of lymphocyte activation	6/30	481/18112	0.000
BP	GO:0002443	leukocyte mediated immunity	6/30	423/18112	0.000
BP	GO:0150063	visual system development	5/30	466/18112	0.001
CC	GO:0031012	extracellular matrix	4/31	408/18418	0.005
CC	GO:0030312	external encapsulating structure	4/31	410/18418	0.005
CC	GO:0043235	receptor complex	4/31	413/18418	0.005
CC	GO:0009897	external side of plasma membrane	4/31	461/18418	0.007
CC	GO:0005765	lysosomal membrane	3/31	216/18418	0.006
MF	GO:0019903	protein phosphatase binding	4/31	185/16650	0.000
MF	GO:0019902	phosphatase binding	4/31	234/16650	0.001
MF	GO:0005126	cytokine receptor binding	4/31	282/16650	0.002
MF	GO:0051087	chaperone binding	3/31	109/16650	0.001
MF	GO:0001223	transcription coactivator binding	2/31	49/16650	0.004
KEGG	rno04630	JAK-STAT signaling pathway	6/17	158/8963	0.000
KEGG	rno04659	Th17 cell differentiation	5/17	104/8963	0.000
KEGG	rno05169	Epstein-Barr virus infection	4/17	226/8963	0.001
KEGG	rno05171	Coronavirus disease -COVID-19	4/17	318/8963	0.003
KEGG	rno04658	Th1 and Th2 cell differentiation	3/17	89/8963	0.001

BP, biological processes; CC, cellular components; MF, molecular functions. KEGG, Kyoto Encyclopedia of Genes and Genomes.

### PPI network demonstrated the seven hub genes from co-DEGs

**Figure 6:**
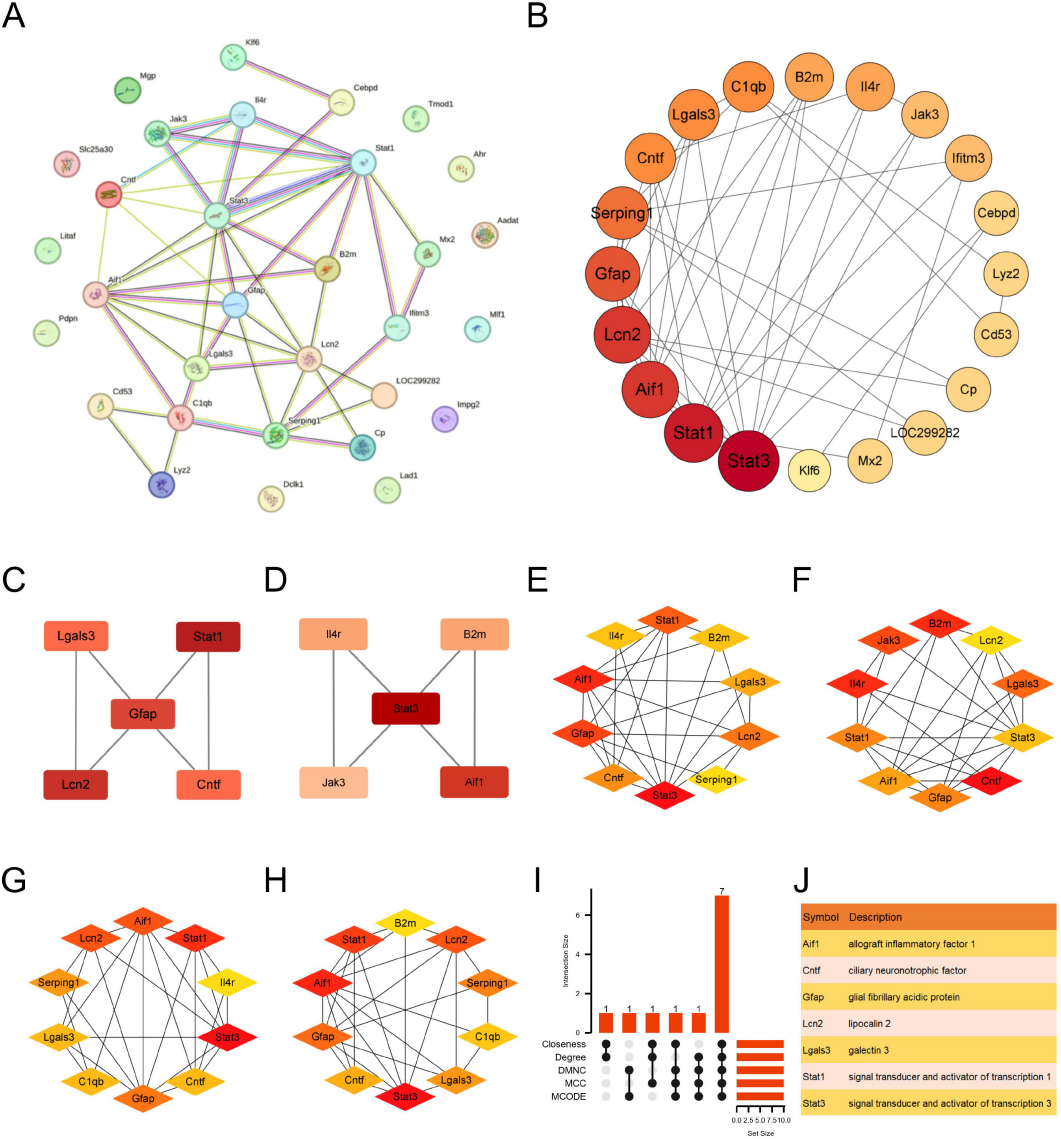
PPI networks analysis results of the co-up DEGs. **(A)** The presentation of PPI network involving the 31 co-up DEGs by utilizing the STRING database. **(B)** The presentation of PPI network for the interacting DEGs visualized by Cytoscape software. **(C, D)** Interaction network of two clusters derived from the MCODE plugin analysis. **(E-H)** Interaction network of the top 10 nodes calculated using the MCC **(E)**, DMNC **(F)**, Degree **(G)**, and Closeness **(H)** algorithms in the cytoHubba plugin. **(I)** Overlapping results of gene intersection derived from the MCODE and cytoHubba algorithms. **(J)** Details of the seven hub genes, including *Aif1, Cntf, Gfap, Lcn2, Lgals3, Stat1*, and *Stat3*.

The PPI network for the 31 co-DEGs was then constructed (**Figures 6A, B**). Two clusters were identified through the MCODE plugin method. Cluster 1 included *Stat1*, *Lcn2*, *Gfap*, *Cntf*, and *Lgals3* (**Figure 6C**), while Cluster 2 included *Stat3*, *Aif1*, *Jak3*, *Il4r*, and *B2m* (**Figure 6D**). The cytoHubba plugin was utilized for screening the top 10 genes by using the Maximum Neighborhood Component (MNC), Degree, and Closeness algorithms (**Figures 6E–H**). Gene intersection was subsequently derived from the MCODE and cytoHubba algorithms (**Figure 6I**). Eventually, seven potential hub genes were identified, including *Aif1*, *Cntf*, *Gfap*, *Lcn2*, *Lgals3*, *Stat1*, and *Stat3* (**Figure 6J**).

**Figure 7:**
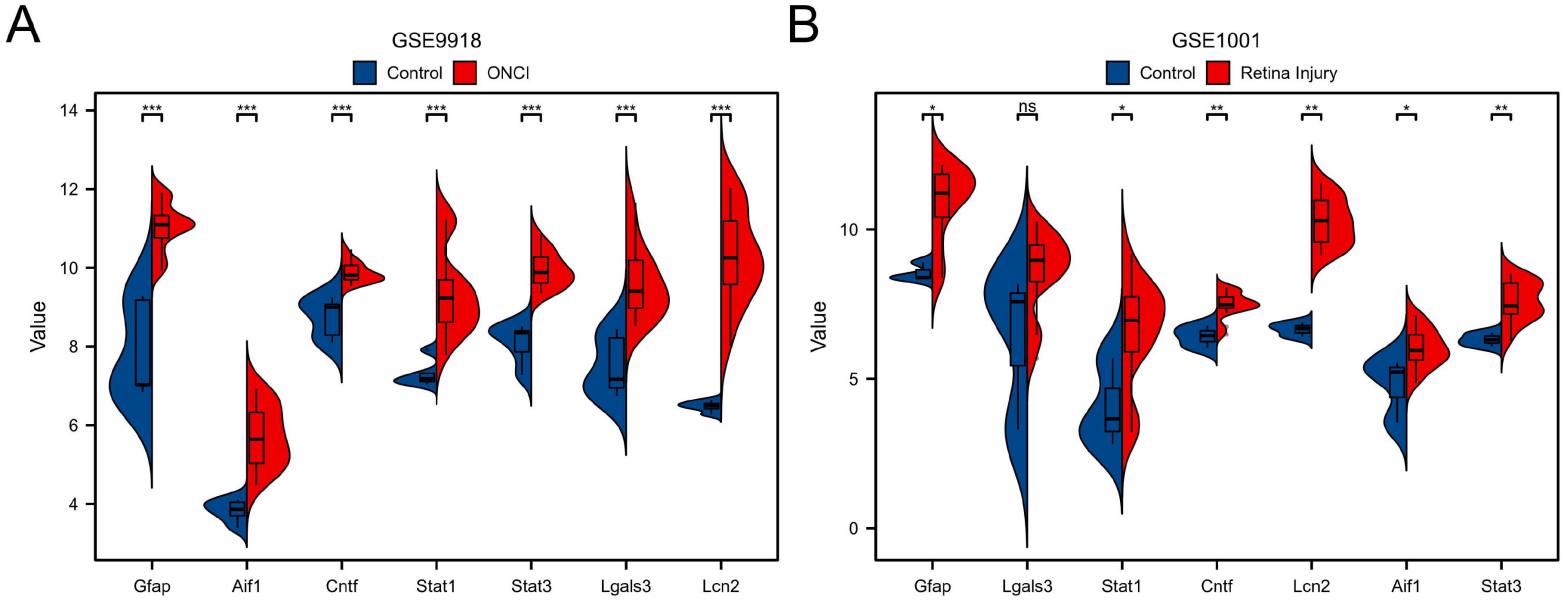
Expression analysis of the predicted hub genes in these two datasets. **(A, B)** Expression patterns and differential significance of the seven hub genes in **(A)** GSE9918 and **(B)** GSE1001. *p < 0.05; **p < 0.01; ***p < 0.001; ns, non-significant.

To confirm whether the expression patterns differ remarkably, we re-analyzed the expression levels of *Aif1*, *Cntf*, *Gfap*, *Lcn2*, *Lgals3*, *Stat1*, and *Stat3* in the GSE9918 and GSE1001 datasets, respectively. In the GSE9918 dataset, all seven potential hub genes showed remarkable differences in their expression levels (*p*<0.001) (**Figure 7A**). In the GSE1001 dataset, *Aif1*, *Cntf*, *Gfap*, *Lcn2*, *Stat1*, and *Stat3* differed significantly (*p*<0.05) in their expression levels. However, *Lgals3* expression level exhibited no significant difference (*p*>0.05) (**Figure 7B**). Thus, *Aif1*, *Cntf*, *Gfap*, *Lcn2*, *Stat1*, and *Stat3* were recognized as hub genes for both datasets.

### Diagnostic verification of the six hub genes within experimental datasets

**Figure 8:**
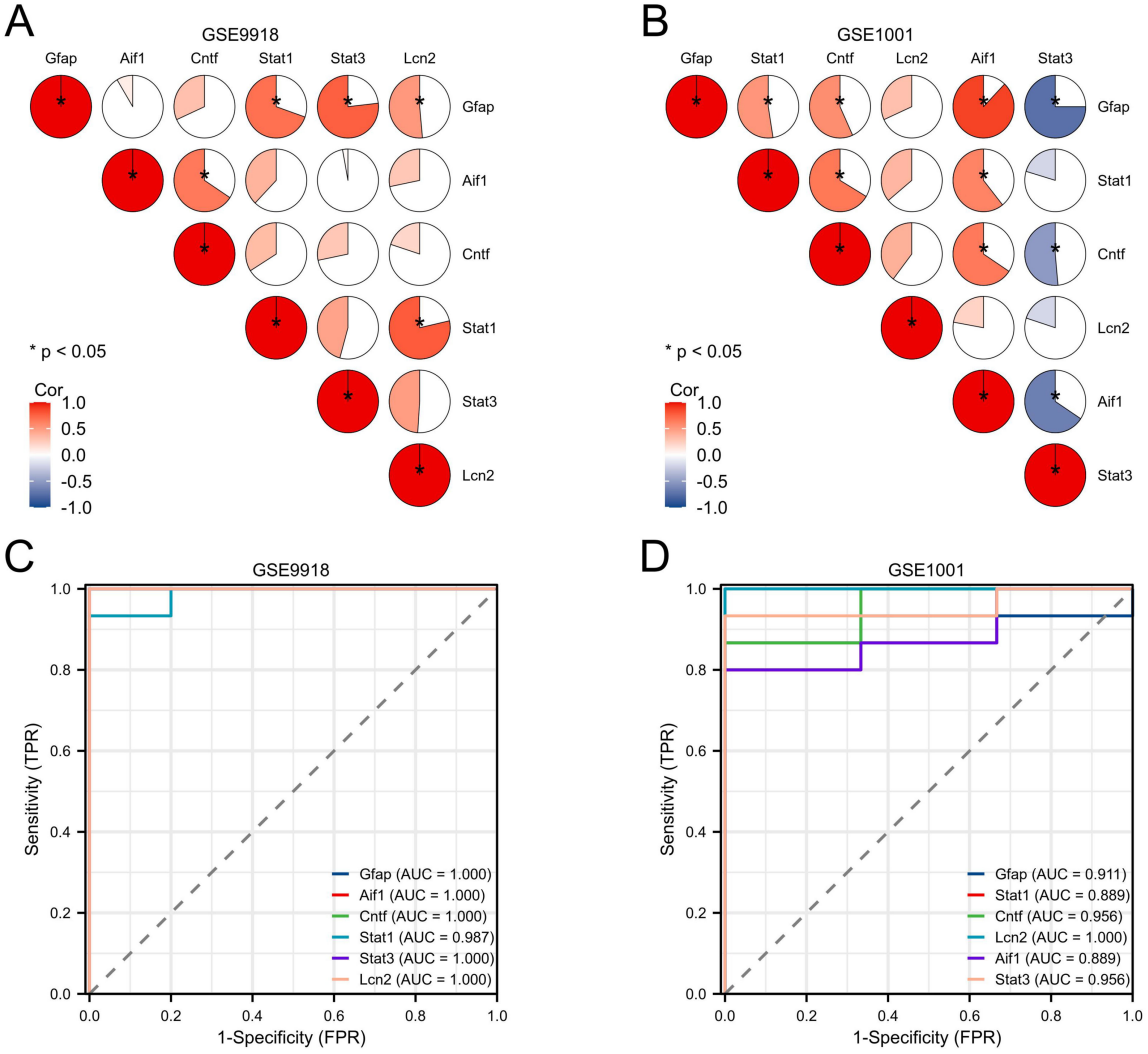
Diagnostic analysis of the hub genes within these two experimental datasets. **(A, B)** Bitmap for correlation analysis of the six hub genes in the GSE9918 **(A)** and GSE1001 **(B)** datasets. * *p*<0.05. **(C, D)** ROC curves for the diagnostic performance of the six hub genes in **(C)** GSE9918 and **(D)** GSE1001. AUC, area under curve.

Next, correlation analysis of the above mentioned six hub genes was performed using Spearman’s statistical method (**Figures 8A, B**). Meanwhile, validation by the ROC curve analysis revealed that *Aif1*, *Cntf*, *Gfap*, *Lcn2*, *Stat1*, and *Stat3* exhibited a remarkable diagnostic value in the GSE9918 and GSE1001 datasets [area under the curve (AUC)>0.9 and 0.8, respectively] (**Figures 8C, D**), thus confirming *Aif1*, *Cntf*, *Gfap*, *Lcn2*, *Stat1*, and *Stat3* as the hub genes with potential diagnostic significance within the experimental animal datasets.

### STAT1 and STAT3 were potential regulators of the hub genes

**Figure 9:**
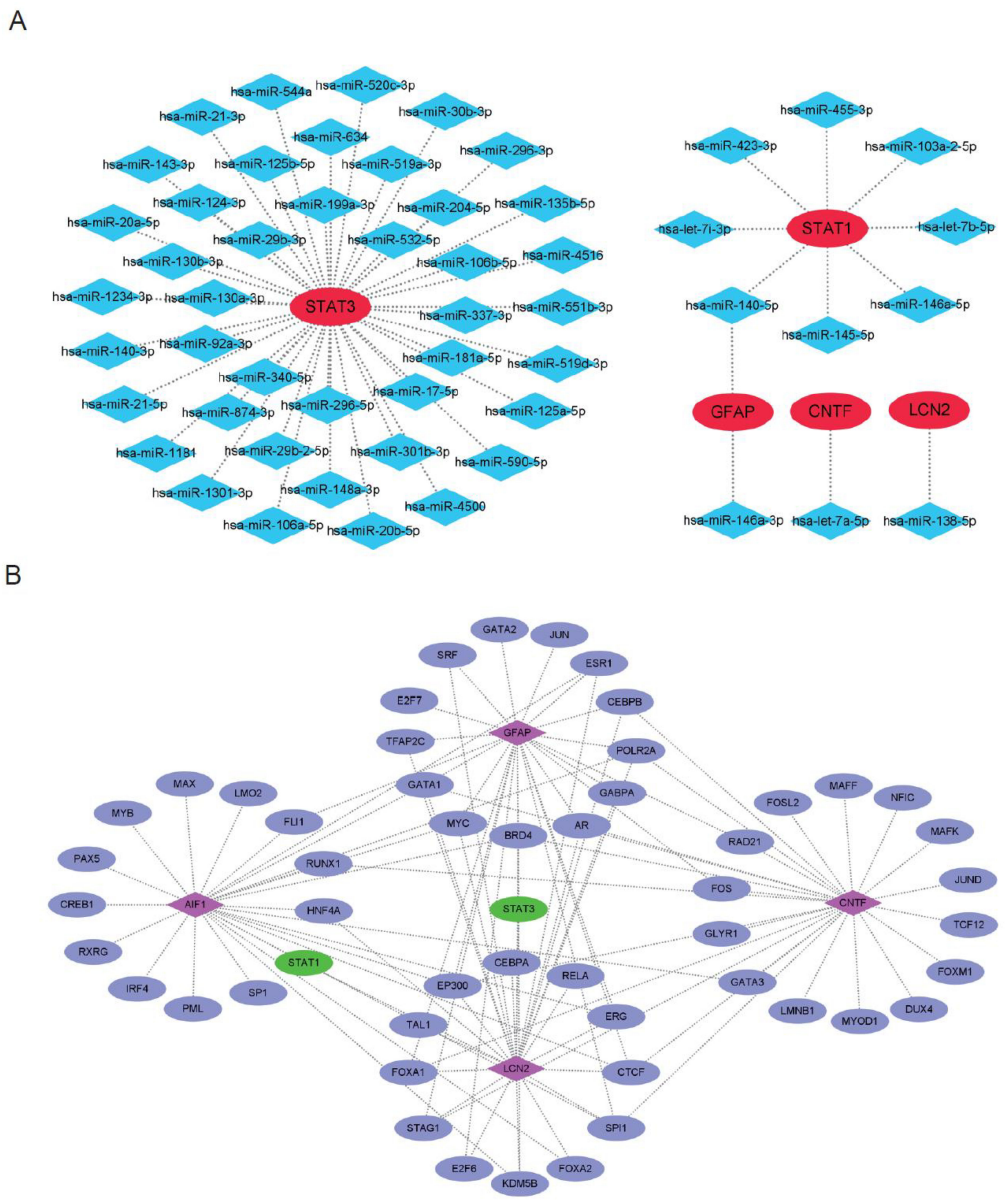
Regulatory network of hub genes by predicting upstream miRNAs and TFs. **(A)** Potential regulatory network showing the correlations between miRNAs and the hub genes. Red circles and blue diamonds represent hub genes and miRNAs, respectively. **(B)** Potential regulatory network showing the correlations between TFs and target genes. Pink diamonds represent target genes, purple circles represent TFs, and green circles represent two crucial TFs.

To identify miRNAs and TFs associated with the hub genes in humans, we first performed homologous transformation of the hub genes from *Rattus norvegicus* to *Homo sapiens* (**Table 4**). Next, by utilizing the miRTarBase, we constructed an interaction network of hub genes-miRNAs, which comprised five genes and 54 miRNAs (**Figure 9A**), including *STAT1* and *STAT3* as TFs. We used the hTFtarget database to explore the TFs regulating *AIF1*, *CNTF*, *GFAP*, and *LCN2* and selected 25 TFs for each target gene to construct an interaction network (**Figure 9B**). The results showed that *STAT1* was related to *AIF1* and *LCN2*, while *STAT3* was linked with *GFAP* and *LCN2* (**Figure 9B**). More details were provided in **Additional File 4**. These results demonstrated that STAT1 and STAT3 may play potential roles in retinal injury by regulating gene expression pattern.

**Table 4:** Homologous transformation of the hub genes between rat and human.

ID	Rat symbol	Rat entrez id	Human symbol	Human entrez id
Aif1	Aif1	29427	AIF1	199
Cntf	Cntf	25707	CNTF	1270
Gfap	Gfap	24387	GFAP	2670
Lcn2	Lcn2	170496	LCN2	3934
Stat1	Stat1	25124	STAT1	6772
Stat3	Stat3	25125	STAT3	6774

## Discussion

Ocular trauma commonly causes vision disorders, often leading to decreased visual acuity, blurred vision, visual field loss, or even blindness. TONI and TRI are two common and closely linked types of ocular injuries. The retina constitutes neurons, blood vessels, and glial cells. RGCs are neurons located in the retina’s innermost layer; they serve as the output cells and transmit visual signals from the retina to the brain (21). The optic nerve comprises RGC axons. Any severe external injuries to the retina or optic nerve can cause degeneration or death of RGCs, resulting in permanent vision damage (22). Although several neuroprotective strategies have been investigated to promote RGC regeneration, such as neurotrophic factor therapy (23, 24), stem cell therapy (25, 26), and gene therapy (27, 28), their capacity for regeneration and functional recovery remains limited. Moreover, the precise cellular and molecular mechanisms of RGC death caused by TONI or TRI are not fully elucidated. Therefore, we investigated differences in gene expression in optic nerve crush injury and retinal injury rat models and identified six hub genes: *Aif1*, *Cntf*, *Gfap*, *Lcn2*, *Stat1*, and *Stat3*, which can be further validated in additional datasets to evaluate their potential as biomarkers or targets for ocular trauma.

Allograft inflammatory factor 1 (AIF1), a calcium-binding protein, can promote macrophage activation and proliferation (29), and is critically involved in the pathological immune and inflammatory responses (30). Microglia, resident macrophages in the central nervous system (CNS), along with oligodendrocytes, neurons, and astrocytes maintain CNS homeostasis and function (31–33). Previous studies have shown that AIF1 functions as a specific microglial activation marker, and it is closely related to neural diseases, including Alzheimer’s disease (34), cerebral infarction (35), and spinal cord injury (36). Microglia are critical components of the retinal and optic nerve and participate in surveilling the neuro environment and regulating retinal homeostasis (37). Abnormally activated microglia may lead to retinal degeneration and loss of RGCs by interfering with normal neural and glial cell functions (38, 39). However, changes in AIF1 expression and regulatory functions in TONI or TRI have not been investigated. According to our results, compared to the control group, AIF1 expression is significantly upregulated in the optic nerve injury or retinal damage groups. Therefore, AIF1 could be a valuable target for ocular trauma research.

Ciliary neurotrophic factor (CNTF), a neurotrophic factor, facilitates the growth, proliferation, and differentiation of neuronal cells, including RGCs. Additionally, CNTF enables injured RGCs to survive and regenerate (40). In the retina, various glial cells, particularly Müller cells, secrete CNTF (21). Behtaj et al. showed that CNTF mediates RGC axon growth in *in vitro* experiments (41). Do Rhee et al. revealed that CNTF influences the metabolic status of the retina by enhancing anabolic activities and aerobic glycolysis (42). Moreover, Blanco et al. showed that the application of CNTF can increase the survival numbers and elongation rate of RGC axons in an optic nerve crush frog model (43). According to our study, CNTF expression was remarkably increased in the ocular trauma models, thus suggesting its potential neuroprotective functions to prevent RGC degeneration.

In mature astrocytes, glial fibrillary acidic protein (GFAP) functions as the major intermediate filament protein (44). Under physiological conditions, GFAP is expressed in astrocytes alone and almost not expressed in Müller cells in the retina. Following injury, GFAP expression in Müller cells shows a reactive increase, thus making it a critical marker for Müller cell activation (45). Hirahara et al. noted increased accumulation of GFAP-positive Müller cells and astrocytes in the retinal layer after TONI (46). Bramblett et al. tested the serum GFAP levels in porcine optic nerve crush models and revealed significantly elevated post-crush GFAP levels (44). Our results demonstrated a similar enhancement in GFAP expression in TONI and TRI groups.

Lipocalin-2 (LCN2), a circulating lipocalin family protein, participates in multiple pathological processes, including inflammation, iron homeostasis, and cell differentiation and migration (47, 48). In the CNS, LCN2 is majorly secreted by activated astrocytes following inflammatory and pathological stimuli (49). LCN2 can modulate astrocyte activation and polarization, thereby potentially amplifying inflammatory response and exacerbating nerve injury (50). Elevated LCN2 levels are linked with various neurological diseases, particularly brain injury, Alzheimer’s disease, spinal cord injury, and ischemic stroke (51–54). However, the functions and expression levels of LCN2 in TONI and TRI have not been explored. We noted that LCN2 expression is remarkably upregulated in ocular traumatic groups, thus suggesting it might function as a potential biomarker for TONI or retinal damage.

STAT1 and STAT3 are the signal transducers and activators of transcription (STAT) family members; they are TFs involved in the JAK-STAT signaling pathway, which is critical for innate and adaptive immune responses and is triggered by several cytokines and interferons (55). This pathway also has a crucial effect on neuroinflammatory diseases and optic neuropathy (56, 57). Zhang et al. exhibited that *Ginkgo biloba* extract attenuates ischemic brain injury by inhibiting astrocytic LCN2 expression and reducing neuroinflammatory response through the JAK2-STAT3 signaling pathway (58). As reported by Wang et al., LCN2 expression and JAK2-STAT3 pathway activation are triggered mutually after spinal cord injury, thereby contributing to the activation of astrocytes and microglia (59). Elsaeidi et al. reported that, following optic nerve injury, IL-6 family cytokines stimulate the JAK-STAT pathway in RGCs and enhance optic nerve regeneration in zebrafish models (60). Chen et al. revealed that the survival of RGCs in zebrafish optic nerve injury models is regulated by the JAK-STAT pathway and innate immune responses (61). Consistently, our study demonstrated that STAT1 and STAT3 are overexpressed in TONI and TRI groups, and the DEGs are mainly enriched in the JAK-STAT signaling pathway, thus revealing that this pathway may participate in regulating inflammation and RGC regeneration in TONI and TRI. In addition, our TF regulatory network analysis showed that STAT1 is related to AIF1 and LCN2, and STAT3 is associated with GFAP and LCN2, thus indicating a close association between the expression of the hub genes and the JAK-STAT signaling pathway.

Meanwhile, there are some limitations of our study. First, because this study mainly involved bioinformatics analysis, *in vivo* experiments or clinical testing are further required to validate expression patterns and potential as biomarkers of these six hub genes. Second, further investigations are warranted to determine the precise molecular regulatory mechanisms of these genes in TONI and TRI. Third, the mutual regulatory effects among the hub genes and their relationship with the JAK-STAT signaling pathway are unclear and warrant further research.

## Conclusion

Our study identified six candidate genes, namely *AIF1*, *CNTF*, *GFAP*, *LCN2*, *STAT1*, and *STAT3*, which may directly or indirectly regulate RGCs regeneration in TONI and TRI. The JAK-STAT signaling pathway may have remarkable involvement in TONI and TRI by regulating neuroinflammation and promoting axonal regeneration. These findings offer novel perspective regarding the cellular and molecular mechanisms underlying TONI and TRI.

## Data Availability

The GSE9918 and GSE1001 datasets analyzed during the current study are available in the GEO database. All data analyzed or generated in this paper have been provided in this article and its **Supplementary Materials**. Web links and URLs are as follows: The GEO database (https://www.ncbi.nlm.nih.gov/geo/). The STRING database (https://cn.string-db.org/). The miRTarBase database (https://mirtarbase.cuhk.edu.cn/). The hTFtarget database (https://guolab.wchscu.cn/hTFtarget/). The JASPAR database (https://jaspar.elixir.no/). The KEGG pathway database (https://www.kegg.jp/kegg/pathway.html).
